# Advances in Resistant Starch Research from Agro-Industrial Waste: A Bibliometric Analysis of Scientific Trends

**DOI:** 10.3390/foods14162815

**Published:** 2025-08-14

**Authors:** Milena A. Saavedra-Cordova, Valeri S. Mosilot-Acosta, Dorila E. Grandez-Yoplac, Segundo G. Chavez, Grobert A. Guadalupe

**Affiliations:** 1 Grupo de Investigación en Seguridad Alimentaria (GISA), Facultad de Ingeniería y Ciencias Agrarias (FICA), Universidad Nacional Toribio Rodríguez de Mendoza de Amazonas, Calle Higos Urco N° 342-350-356—Calle Universitaria N° 304, Chachapoyas 01001, Peru; 7534173092@untrm.edu.pe (M.A.S.-C.); 7322666192@untrm.edu.pe (V.S.M.-A.); dorila.grandez@untrm.edu.pe (D.E.G.-Y.); grobert.guadalupe@untrm.edu.pe (G.A.G.); 2 Instituto de Investigación, Innovación y Desarrollo Para el Sector Agrario y Agroindustrial (IIDAA), Universidad Nacional Toribio Rodríguez de Mendoza de Amazonas, Calle Higos Urco N° 342-350-356—Calle Universitaria N° 304, Chachapoyas 01001, Peru; 3 Instituto Universitario de Ingeniería de Alimentos Food-UPV, Universitat Politècnica de València, Camino de Vera s/n, 46022 Valencia, Spain

**Keywords:** food safety, risk assessment, reuse, sustainability, functional food

## Abstract

This study comprehensively analysed the scientific production of the extraction, characterisation, and toxicological risk of resistant starches obtained from agro-industrial by-products. Articles indexed in the Scopus database between 2015 and 2025 were analysed. The results showed a progressive increase in publications led by Chinese institutions, the most notable being Jiangnan University. Most of the studies were published in high-impact journals, with the International Journal of Biological Macromolecules standing out, followed by Carbohydrate Polymers and Food Chemistry, all in the first quartile. Most studies focused on extraction methods (physical, chemical, and mechanical) and starch characterisation (morphological, structural, molecular, physicochemical, and functional). Emerging trends are directed towards innovative applications such as functional foods. However, the risks associated with contaminants in reusing agro-industrial by-products have not been adequately explored, showing an important gap in the current scientific literature. In this context, future research should focus on evaluating toxicological risks derived from these processes, considering the presence and behaviour of heavy metals, pesticides, and mycotoxins, as well as the possible migration of chemical compounds generated during extraction.

## 1. Introduction

Resistant starches (RSs) have gained great attention in nutrition and the food industry due to their functional properties and potential health benefits [1]. Unlike conventional starches, RSs are not digested in the small intestine and reach the colon; they are fermented by intestinal microbiota, promoting the production of short-chain fatty acids [2]. These compounds are associated with improved intestinal and metabolic health [3]. In addition, multiple studies have shown that RSs contribute to the regulation of postprandial glucose and insulin levels, as well as to the prevention and control of chronic diseases such as obesity, type 2 diabetes, cardiovascular disease, certain types of cancer, and inflammatory diseases, thanks to their ability to reduce oxidative stress and inflammation [4,5,6]. In fact, in the scientific literature we find the following five recognised types of RS, according to their structure and origin: RS I (physically inaccessible), RS II (native granular), RS III (retrograde), RS IV (chemically modified), and RS V (amylose-lipid complexes) [7]. On the other hand, the shape of starch crystals is fundamental to their classification; there are basically starches with hexagonal (B-type) and orthorhombic (A-type) structures [8].

In this context, obtaining RSs from agro-industrial by-products or residues like banana pseudostem with RS type III [9], cassava by-products with 12% RS type I [10], Guinea arrowroot with RS types III and IV [11], and mango seeds with RS type II [12] represents a sustainable and functional alternative thanks to the use of carbohydrates, proteins, fibres, and bioactive compounds such as phenolic compounds, carotenoids, and antioxidants, with potential technological applications in food production [13,14,15]. The valorisation of these by-products, driven by the circular economy, allows their incorporation as functional raw materials, reducing the environmental impact derived from their disposal and lowering disposal costs [16,17,18].

However, the incorporation of by-products in food for human consumption also entails certain risks, such as microbial contamination of foodstuffs [19] and the presence of mycotoxins [20] and azetidine-2-carboxylic acid (a potentially toxic non-protein amino acid) [21]. In addition, one of the main challenges in the use of agro-industrial residues is the possible presence of pesticide residues, which pose acute and chronic risks to human health [22]. Despite these challenges, the use of agro-industrial waste remains a promising avenue for the development of functional ingredients such as RSs, provided that it is accompanied by adequate quality control and food safety [23].

Previous studies [9,10,11] on resistant starches have focused on highlighting their functional properties and health benefits. However, there is a gap in the literature on the integration between the benefits and potential risks related to the extraction of resistant starches from agro-industrial by-products, such as the presence of mycotoxins, heavy metals, naturally occurring toxic compounds, or pesticide residues. In this context, a comprehensive literature review is a key tool to identify the main trends and gaps in research and to assess whether these critical aspects are being adequately considered. Therefore, this study aims to analyse scientific production related to the extraction, characterisation, and toxicological risk of RSs derived from agro-industrial by-products, identifying emerging areas, gaps in knowledge, and the level of attention given to potential risks to human health and food safety.

## 2. Materials and Methods

### 2.1. Literature Selection Criteria

**Figure 1:**
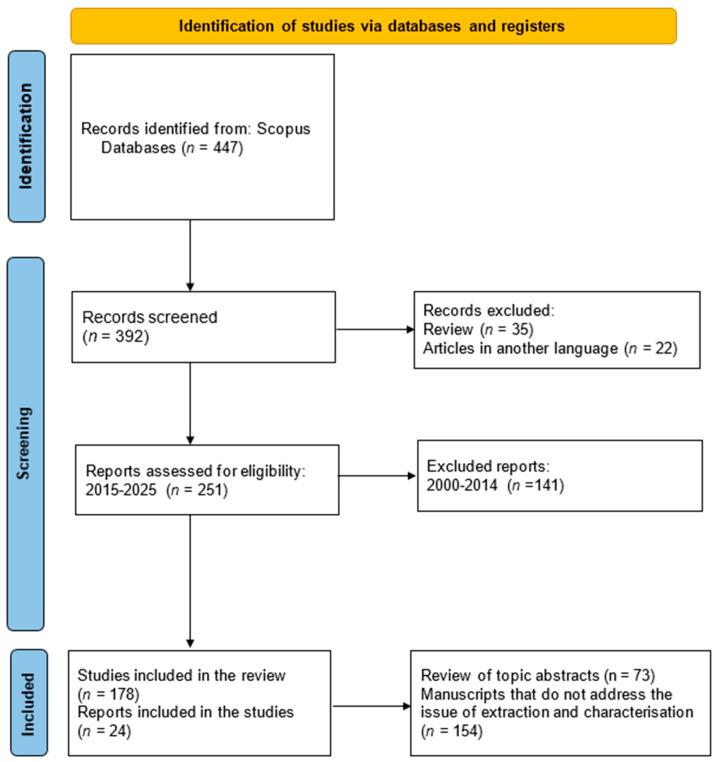
PRISMA flowchart representing the systematic process of the literature search and selection (accessed on 26 March 2025).

A comprehensive literature review was conducted using the PRISMA (Preferred Reporting Items for Systematic Reviews and Meta-Analyses) framework to ensure transparency and scientific quality in the review process [24], following the recommendations of Xiao and Watson [25]. For the literature search, the Scopus database was selected for its broad research coverage, indexing quality, and advanced bibliometric analysis tools [26]. The search criteria filtered titles, abstracts, and keywords, including the following words: resistant AND starch AND by-products OR waste OR residues. These articles were then reviewed to confirm their suitability for inclusion (Figure 1). Only studies that were written in English, available in full text, and in the final stage of publication were included. Papers prior to 2015 were excluded, as well as reviews and articles in a language other than English. Initially, 447 papers were identified. During the screening phase, 35 reviews and 22 articles in other languages were excluded, leaving 392 papers for evaluation. In the eligibility phase, a filter was applied for the year of publication (2015 to 26 March 2025). After reviewing the abstracts and thematic relevance, 178 articles were selected for the study. Of these, 24 studies met all criteria and are included in Section 4.

### 2.2. Data Synthesis and Analysis

The processing and bibliometric analyses of the information were carried out following the recommendations of Guadalupe et al. [27]. The documents were exported in .csv format and analysed using the free software R (v 4.4.1) combined with the Bibliometrix package (version 4.3.3) [28].

A descriptive synthesis was also carried out by reviewing the key terms to summarise the information from the selected studies. The study findings were documented, synthesised, and reviewed to ensure an accurate interpretation. The analysed results are presented in the tables and figures in the Section 3 and Section 4.

## 3. Bibliometric Analysis

### 3.1. Annual Scientific Production

The studies of RSs associated with agri-food by-products between 2015 and 2020 had an irregular growth, ranging from 7 to 19 publications per year. From 2021 to 2024, there was a significant increase in publications. In 2024, the trend reached its peak, with a record 51 publications on the topic, highlighting the growing interest of researchers in the subject. In the months from January to March 2025, 12 publications were produced (see Figure S1).

### 3.2. Production by Country

**Figure 2:**
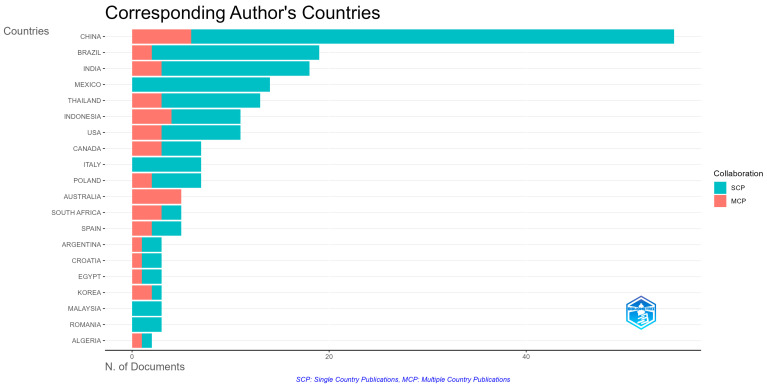
Countries of the authors with the highest scientific production on the subject. SCP: single-country publication; MCP: multiple-country publication.

In the production by country analysis, China stands out in first place, showing a greater contribution that far exceeds other countries. It is followed by countries such as Brazil and India, which, like China, reflect co-authorships with researchers from different countries. Mexico also appears in this list; however, this country shows co-authorships with institutions from the same country, i.e., they have no international collaborations. The following countries are also identified in decreasing order: Thailand, Indonesia, USA, Canada, Italy, Poland, Australia, South Africa, Spain, Argentina, Croatia, Egypt, Korea, Malaysia, Romania, and Algeria. It is important to mention that Italy, Malaysia, and Romania are characterised by the fact that their collaborations are only national, unlike the rest of the countries, which have international links (Figure 2). It should be clarified that numbers do not always imply effective collaboration or efficient production in terms of scientific product quality. More in-depth analyses could reveal more detailed information.

### 3.3. Author Contributions

The leading author is Chinese researcher Li Y., with eight publications, followed by his compatriot Li X., with a total of seven publications (see Figure S2). This indicates a dominant presence of Chinese authors in this field of research. On the other hand, authors such as Amornsakchai T., Chen Y., Hu X., Wongsagonsup R., and Zhang Y. are positioned in the third position of the authorship list, with a total of five publications each. Finally, authors such as Heredia-Olea E., Jin Z., and Smith S.M. have a lower participation, with four publications each. Nevertheless, their participation remains equally relevant to the development of knowledge in this field of study. All publications considered in the ranking are about resistant starches.

### 3.4. Contribution of Journals

**Figure 3:**
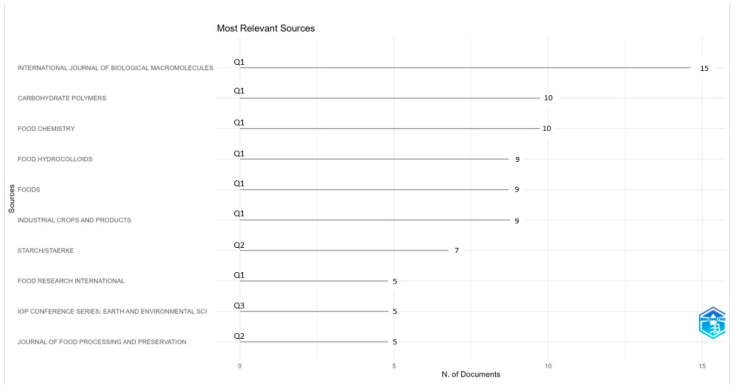
Main journals with publications related to the topic. Q1: 1st quartile; Q2: 2nd quartile; Q3: 3rd quartile.

Figure 3 shows the journals with the highest number of publications related to the topic. The sources highlighted mainly belong to the first (Q1) and second (Q2) quartile according to the Scimago Journal & Country Rank (SJR) for the year 2024 (SJR, 2025), which reflects a high impact and scientific quality. The International Journal of Biological Macromolecules tops the list with 15 publications, consolidating its position as the most influential source in the field. It is followed by Carbohydrate Polymers and Food Chemistry, both with 10 publications, then Food Hydrocolloids, Foods, and Industrial Crops and Products, with 9 publications each. These six journals are ranked in the first quartile, which supports the strength and relevance of their research. On the other hand, sources such as Starch/Stärke (Q2), Food Research International (Q1), IOP Conference Series: Earth and Environmental Science (Q3), and Journal of Food Processing and Preservation (Q2) present a smaller number of papers (between three and seven) but are still relevant in the field of resistant starches.

### 3.5. Contribution of Affiliations

Figure S3 shows the academic institutions with the highest scientific production in the study of RSs from agro-industrial by-products. There is a marked difference in the number of publications among the listed universities. Jiangnan University stands out as the most productive institution, with a total of 30 publications, representing a clear concentration of knowledge at this university. It is followed by Qilu University of Technology (Shandong Academy of Agricultural Sciences), with 12 publications, and the University of Zagreb, with 10. A second group of universities has between eight and nine publications, including Mahidol University, South China University of Technology, and Universidade de São Paulo, indicating an active but less predominant participation compared with Jiangnan University. At the lower end of the graph, the institutions with the lowest scientific output are the National University of Singapore and Wageningen University and Research, both with four publications. Although their contribution is limited in quantity, their presence suggests interest in the topic at the global level. Figure S3 allows us to identify that the study of resistant starches in by-products has captured the attention of universities from different continents, especially led by Asian institutions.

### 3.6. Keyword Analysis

**Figure 4:**
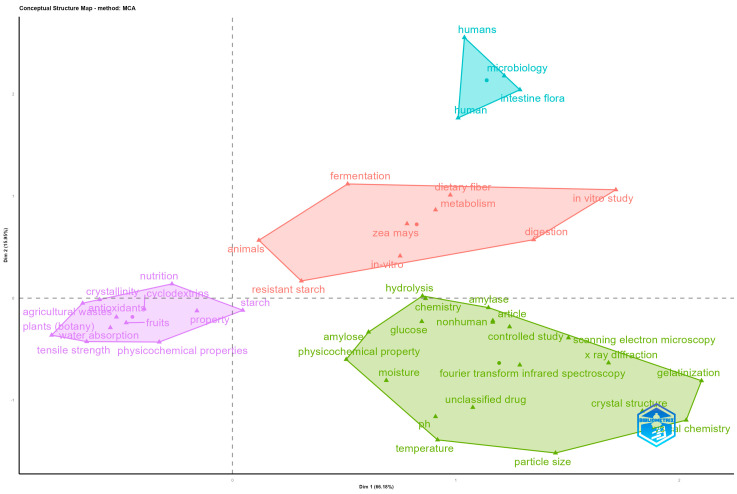
Factor correspondence analysis of keywords.

From the point of view of the variety of concepts handled in the works related to the study of RSs, their properties, and functional applications, Figure 4 clearly shows four differentiated clusters that group together the main thematic lines addressed in the literature analysed. At the bottom right of the graph is the cluster with the highest number of associated terms, including amylose, physicochemical property, temperature, moisture, crystal structure, X-ray diffraction, and scanning electron microscopy. These items are related to studies focusing on the structural and physicochemical characterisation of starch and its derivatives, using advanced analytical tools such as spectroscopy, X-ray diffraction, and electron microscopy. This thematic line reflects a focused approach to the technical and molecular understanding of resistant starches and its functional properties.

Towards the left side of the graph is the cluster formed by terms such as agricultural waste, fruits, plants (botany), antioxidants, water absorption, and nutrition. This cluster is linked to research that addresses the use of plant by-products and agro-industrial waste for the development of ingredients or functional foods, highlighting their nutritional value, their reduced environmental impact, and their potential as a source of antioxidants and dietary fibre.

In the centre of the graph, shifted towards the top, is a cluster clearly linked to studies on digestion and metabolism. It groups terms such as resistant starch, fermentation, dietary fibre, in vitro study, animals, zea mays, and digestion. This group suggests a line of research focused on evaluating the behaviour of resistant starch during digestive transit, its fermentation in the colon, and its impact on metabolic health in both animal models and in vitro studies.

Finally, at the top of the map, there is a small cluster grouping terms such as humans, microbiology, intestinal flora, and human. This directly relates to research aimed at understanding the interaction of resistant starch with the human gut microbiota, as well as its role as a prebiotic and its implications for digestive health.

## 4. Extraction and Characterisation of RSs from By-Products

Of the 178 studies included in the review, 24 of them address the extraction and characterisation of RSs from agro-industrial by-products. Table 1 summarises the main food by-products used as raw materials for the extraction of RSs, as well as the most relevant characteristics of the starch obtained and the extraction methods applied. A wide diversity is observed in terms of the by-product used, the extraction procedure, and the type and characterisation of the RS obtained. This diversity reflects the potential for valorisation of agricultural by-products in different industries.

### 4.1. Agro-Industrial By-Products as a Source of RS

Agro-industrial by-products have been investigated as alternative sources of RSs due to their availability, low cost, and functional potential. Among the most studied are tropical fruit residues (banana peel, passion fruit, cocoa, melon, and mango), stems (pineapple, banana, and cassava), seeds (jackfruit, guava, and mango), cereals and pseudocereals (rice, sorghum, and tiger nut), and tubers such as Guinea arrowroot. These residues have been shown to contain variable proportions of RSs, mostly types RS1, RS2, and RS3, with functional properties such as antioxidant capacities, hypoglycaemic effects, and prebiotic activity [37,44,45,46,49]. Particularly noteworthy are the cases of banana pseudostem and arrowroot, where RS types RS3 and RS4 were identified, associated with retrogradation and thermal modification processes [9,11]. In the case of jackfruit and mango seeds, type RS2 starches were identified, with high crystallinity and enzymatic resistance [12]. This underutilised plant diversity represents a promising avenue towards sustainable food systems, with applications in functional foods and personalised nutrition.

### 4.2. Extraction Techniques Used for the Recovery of RSs

The extraction techniques applied varied according to the plant matrix, and included mechanical, physical, chemical, and combined methods. Mechanical techniques such as milling, soaking, and centrifugation were widely used for fresh by-products such as banana pseudostem, pineapple stalk, or jackfruit seeds [9]. Physical–chemical methods were preferred for residues with more complex structures, such as banana peel, mango seeds, and cooked rice, where heat treatments (autoclaving), alkaline treatments (NaOH), or modifications with octenylsuccinic anhydride (OSA) were applied [29,38]. More sophisticated methods, such as the use of enzyme kits (Megazyme), were employed to quantify RSs in cassava, over-ripe plantain, and passion fruit [10,41]. The selection of the type of extraction directly affects the yield, functionality, and morphology of the starch obtained. Moreover, some methods, such as removal with sodium metabisulfite or alkaline bleaching, facilitate obtaining purified fractions with less structural damage [30]. Consequently, the resistant starch recovery technique required depends on the structure of the source and the type of resistant starch desired.

### 4.3. Characterisation of RSs

The characterisation of the obtained resistant starches included physicochemical, morphological, structural, thermal, and functional techniques, allowing the evaluation of their technological and nutritional properties. Amylose content was a widely reported variable, with values ranging from 26.56 to 38.34%, influencing RS formation and stability [30]. Thermal techniques such as differential scanning calorimetry (DSC) revealed high gelatinisation temperatures (up to 82.39 °C) and significant enthalpies (>19 J/g), such as in pineapple stems and mango seeds, respectively [12]. Morphological observations by electron microscopy identified starch granules with polyhedral, oval, rough, or elongated shapes, which correlate with their digestibility and functional behaviour [35]. Structurally, type A (native starch), type B (retrograded), and type V (lipid-forming) crystallisation patterns were identified as being key for RS classification [9]. In terms of functional properties, high water retention, a low glycaemic index, swelling power, and an antioxidant capacity were reported, particularly in roots such as turmeric and the peels of cocoa, melon, and grape [44,46]. However, standardisation of analytical methodologies and a greater emphasis on the assessment of contaminants or potential toxicological risks associated with agro-industrial residues are still required, as has been noted by recent research focusing on food safety and exposure to heavy metals [50]. Although RS characterisation techniques are practically standard, it is suggested that attempting to identify and quantify the presence of unwanted compounds, usually present in the source of by-products, is required.

## 5. Health Benefits and Risks of RSs from By-Products

Resistant starches, especially those obtained or modified from agro-industrial by-products, have gained relevance as functional ingredients for their ability to promote beneficial effects on human health. Their chemical structure and resistance to digestion allow for differentiated physiological effects related to their type (RS1–RS5), plant origin, and methods of extraction or modification [51].

One of the most outstanding benefits of RSs is their action as a fermentable fibre. Resisting digestion in the small intestine, they reach the colon, where they serve as a substrate for intestinal microbiota, stimulating the growth of beneficial bacteria such as Bifidobacterium and Lactobacillus [7,52]. This process generates short-chain fatty acids, in particular butyrate, known for their anti-inflammatory, colonic-epithelium-protective properties and their ability to reduce the risk of inflammatory diseases and colorectal cancer [53].

In addition, RSs reduce starch digestibility and the glycaemic index (GI) of foods. Several studies have shown their effect on lowering postprandial glucose and insulin spikes, which makes them useful in the management of type 2 diabetes and metabolic syndrome [54,55]. There is also evidence of increased satiety through the release of appetite-regulating peptides such as GLP-1 and PYY, as well as a possible improvement in lipid and energy metabolism, particularly in overweight people [56].

Regarding the lipid profile, RSs contribute to lowering serum and LDL cholesterol levels. This is achieved through mechanisms such as reduced bile acid reabsorption and the production of propionate in the colon, which inhibits hepatic cholesterol synthesis [57,58]. When RSs are extracted from matrices rich in phenolic compounds, they can retain bioactive fractions that provide indirect antioxidant activity, contributing to reducing oxidative stress and preventing chronic diseases [59].

Overall, in vitro and in vivo studies have shown that RSs can improve digestive, metabolic, and cardiovascular health [30,35,60], aiding in the prevention of diseases such as diabetes, dyslipidaemias, obesity, colon cancer, and chronic inflammatory disorders [9,45,49,61,62,63].

However, despite these benefits, safety concerns arise when RSs are obtained from agro-industrial waste. Currently, there are no studies that directly assess the presence of contaminants in extracted RSs; however, the presence of toxic compounds in plant by-products used as raw materials has been reported.

Potential risks include heavy metals such as lead, cadmium, mercury, or arsenic, which can accumulate in plant residues due to environmental pollution or the intensive use of agrochemicals [64,65]. These metals may remain in the final product if appropriate purification processes are not applied, posing risks to organs such as the liver, kidneys, and nervous system [66,67].

Residues of pesticides, chlorine, and chemically modifying compounds such as hypochlorites or peroxides have also been documented, and must be rigorously removed to avoid acute or chronic toxicity [68,69]. Similarly, mycotoxins such as aflatoxins, fumonisins, and deoxynivalenol (DON), present in improperly stored waste, pose significant carcinogenic and immunosuppressive risks [70].

**Figure 5:**
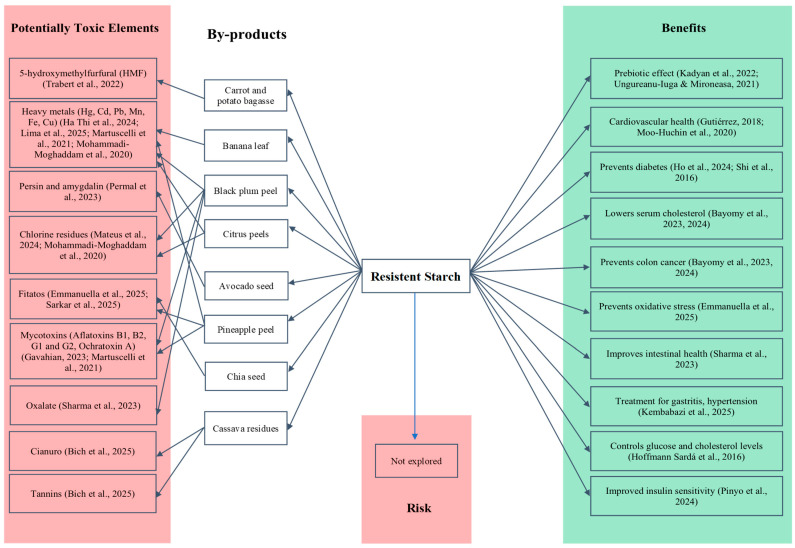
Duality of resistant starch from agro-industrial by-products: functional benefits vs. potential toxicological risks (Reference: [9,30,35,45,48,49,60,61,62,63,64,65,66,67,68,69,70,71,72,73]).

In addition, anti-nutritional components such as phytates, oxalates, tannins, or saponins can interfere with the absorption of essential minerals or lead to adverse gastrointestinal effects [62,63,71,72,73] (Figure 5). In the case of chemical extraction of resistant starches, reagent residues must be strictly monitored as they may induce mutagenic or carcinogenic effects if permitted levels are exceeded [74,75].

Table 2 summarises the key formulas and metrics used in the quantitative assessment of risk from exposure to potentially toxic elements (PTEs). These metrics vary depending on the nature of the pollutant and the type of toxicological effect it may induce. For compounds with non-genotoxic effects and a defined threshold, the hazard quotient (HQ) is commonly used, which is calculated as the ratio of the estimated exposure to a reference value (RfD) below which no adverse effects are anticipated. An HQ value of less than one indicates a negligible toxicological concern [76].

In the case of genotoxic or carcinogenic substances, the margin of exposure (MOE) is used as an alternative metric. This tool is recommended by the European Food Safety Authority (EFSA) and the Joint FAO/WHO Expert Committee on Food Additives (JECFA) as a criterion for prioritising public health risks [81]. The MOE is determined by comparing a toxicological benchmark—usually the modelling-based lowest observed adverse effect level (BMDL_10_ or BMDL_01_)—with the estimated human exposure dose. MOE values ≥ 10,000 are considered to be of low concern for both neoplastic and non-neoplastic effects [82].

Cancer risk (CR) is used to estimate the probability of developing carcinogenic effects after continuous exposure to genotoxic agents [78]. This value is calculated by multiplying chronic exposure by the slope factor derived from dose–response studies. According to international guidelines, a risk below 1.0 × 10^−6^ is considered to be negligible, between 1.0 × 10^−6^ and 1.0 × 10^−4^ represents an intermediate risk zone that may require management measures, and values above 1.0 × 10^−4^ are categorised as unacceptable from a public health perspective [82].

## 6. Trends and Suggestions for Future Research

**Figure 6:**
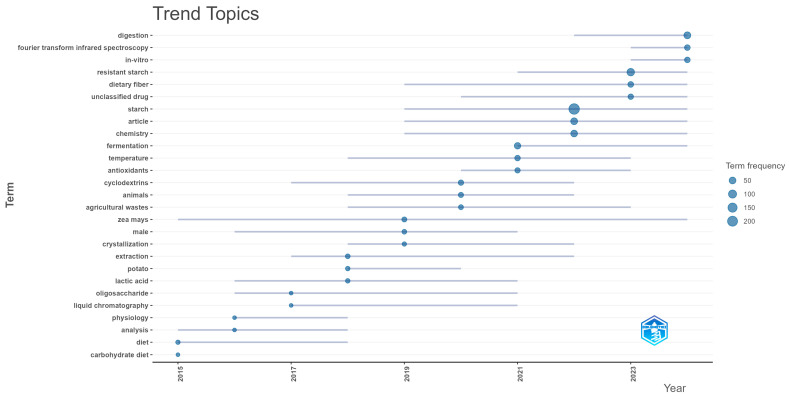
Trend topics of the publications studied from 2015 to 2025 included in the bibliometric analysis.

Figure 6 provides a detailed overview of the recently studied trending terms. In 2015, studies mainly focused on dietary aspects, with a low volume of publications. That year, research on butyrate production during prolonged fermentation of high-amylose corn starch residues pre-digested with human faeces was reported, showing an increase in butyrate production [83].

During 2016, predominant terms such as analysis and physiology were identified. In 2017, interest in the term oligosaccharide increased, and others, such as liquid chromatography, also emerged. In this period, a study was conducted on hydroxyl-radical-induced starch oxidation using electrospray mass spectrometry, where structural changes in amylose and amylopectin were evidenced, including the formation of functional groups such as keto-, hydroxyl-, and hydroperoxides, as well as oxidative ring cleavages [84].

In 2018, growth was observed in research related to lactic acid, potatoes, and extraction. Of particular note was the development of pH-sensitive edible films made from Guinea maranta starch and wine residues. The results indicated that these residues generated cross-linking in the thermoplastic starch matrix, favouring the formation of resistant starch type 4 (RS4) and, to a lesser extent, type 3 (RS3) [11].

In 2019, greater relevance was identified for Zea mays, males, and crystallisation. In 2020, the presence of studies related to cyclodextrins, animals, and agricultural waste increased. One study evaluated the effect of citric acid pretreatment and different drying temperatures on the quality of flours obtained from green plantain pulp and peel [85]. By 2021, there was a significant increase in the number of publications, with the terms fermentation, temperature, and antioxidants standing out. Representative studies include the use of discarded banana pulp for the production of flour with high levels of starch and amylose [86]. In 2022, the term starch was consolidated with chemistry. The optimisation of ultrasound-assisted enzymatic extraction of resistant starch from green plantain peel was reported [87].

During 2023, the most frequent topics were resistant starch, dietary fibre, and unclassified drugs. A study on ginger starch was conducted in which the effect of different freeze–thaw cycles on its properties was evaluated. It was observed that as the number of cycles increased, the content of resistant starch, amylose, and total starch increased [88]. Furthermore in 2024, the terms digestion, Fourier transform infrared spectroscopy, and in vitro were highlighted. Relevant studies include the production of noodles from a mixture of rice flour and pineapple stalk starch, an agricultural by-product, showing favourable functional properties [30]. Finally, the data analysed in our study extended only up to March 2025; therefore, the trend themes are not presented in Figure 6. Nevertheless, we were able to identify several noteworthy studies, such as that of Yüce et al. [89], which involved a risk assessment of essential oils extracted from orange peel.

On the basis of the analysis carried out in this review, the following suggestions are made to guide future lines of research:

(a) Identify and quantify potentially toxic elements in starches extracted from agro-industrial by-products. Hazards in food are often present in very low concentrations, so analytical techniques could also be improved for rapid and accurate detection, especially to quantify emerging hazards.
(b) Assess the sustainability of the reuse of agro-industrial by-products in the extraction of resistant starches and their application in the food industry.
(c) Assess the toxicological risk of the extraction of resistant starches from agro-industrial by-products by investigating dose–response models based on epidemiological studies, exposure, weight, and age. In addition, use probabilistic models to address uncertainties and make informed risk decisions.
(d) Integrate risk–benefit research to promote informed consumption, prevent hazards, and protect consumer health.

## 7. Conclusions

This bibliometric study shows that, since 2015, there has been a progressive growth in resistant starch research, led by Chinese institutions. Most of the studies were published in high-impact journals, with the International Journal of Biological Macromolecules standing out, followed by Carbohydrate Polymers and Food Chemistry, all in the first quartile. The studies focused on extraction methods and starch characterisation.

New trends are moving towards innovative applications such as functional foods, considering that resistant starches offer great health benefits.

Resistant starches derived from agro-industrial by-products have a promising functional profile but their safe application in functional foods, supplements, or pharmaceuticals requires the development of standardised protocols for extraction, purification, and toxicological evaluation to ensure their safety and sustainability.

The risks associated with contaminants in the reuse of agro-industrial by-products have not yet been explored, which shows an important gap in the current scientific literature. In this context, future research should focus on the assessment of toxicological risks arising from these processes, considering the presence of potentially toxic elements, as well as the possible migration of chemical compounds generated during extraction.

## Figures and Tables

**Table 1:** Agro-industrial by-products used to obtain resistant starch: extraction methods, characterisation, and type of RS identified.

By-Product	Extraction	Characterisation	Characteristics Assessed	RS Type	Reference
Pineapple stalk	Mechanical	Physicochemical Morphological Thermal Structural	High amylose content (34.4%) High gelatinisation temperature High enthalpy of gelatinisation (19.4 J/g) Particle size (median of 9.69 µm) Type A crystalline structure	N.S.	[29]
	Chemical	Functional Physicochemical Morphological Structural Thermal	High amylose content (30.56%) Higher proportion of slow-digesting starch and RS Larger granules than rice starch Crystalline structure of type A (in its pure form); in noodles, a mixture of types B and V due to retrogradation Higher thermal stability	N.S.	[30]
	Physical	Morphological Thermal Physicochemical	High amylose content (30.82%) High gelatinisation temperature High content of slowly digested starch (SDS) High RS content Low paste viscosity	N.S.	[31]
Pea waste	Chemical	Morphological Physicochemical	Large and variable particle size Amorphous structure High RS content Slow digestion	N.S.	[32]
Jackfruit seeds (*Artocarpus heterophyllu*)	Mechanical/chemical	Physicochemical Structural Thermal	High percentage of RS Smooth, round, or bell-shaped granules (5–11 μm) High thermal stability	RS2	[33]
	Mechanical/chemical	Structural Morphological Thermal	Type A crystalline structure Variations in granule shape and size Variations in thermal properties (gelatinisation temperature and enthalpy) High amylose content (26.56–38.34%)	N.S.	[34]
Cooked rice waste	Physical/chemical	Thermal Morphological Physicochemical Structural	Changes in gelatinisation temperatures (To, Tp, and Tc) and enthalpy (ΔH) by DSC Polyhedral granules with rough surfaces observed by SEM Changes in chemical structure detected by FTIR Starch, protein, and moisture contents	N.S.	[35]
Tiger nut flour oil *(Cyperus esculentus)*	Physical/chemical	Physicochemical Morphological Thermal	Higher amylose content than native starch Higher swelling power Lower gelatinisation temperature after modification with OSA Unique microstructure (thin fibres and oblate structure) after modification with OSA	N.S.	[36]
Banana pseudostem	Mechanical/physical	Morphological Structural Physicochemical	Native starch has irregular granules and a B-type crystalline structure	RS3	[9]
Cassava by-products (root, stem, and cassava bagasse)	Physical/mechanical/chemical	Physicochemical Functional	Yield of 12% RS Water retention Functional properties (prebiotic effects)	RS1	[10]
	Physical/mechanical	Physicochemical Morphological Functional	RS content Amylose content Solubility Moisture Swelling Granule shape Digestibility Freeze–thaw stability	N.S.	[37]
Orange bagasse	Chemical	Physicochemical Functional	Glucose, fructose, saccharose, and pectin Small amounts of starch Digestion Capacity for fibre supplementation	N.S.	[10]
Maracuyá shell	Chemical	Physicochemical Functional	Pectin and hemicellulose Starch content Fibre supplementation Digestion Low level of methylesterification	N.S.	[10]
Plantain mask	Physical/mechanical/chemical	Morphological Physicochemical Thermal Functional	Single or separate granules; elongated, cylindrical shape Starch source pH varies between 4 and 7 Moisture content of 6.06 ± 0.83% Gelatinisation temperature of 59.75 °C (DSC) Gelatinised starch Amylose and amylopectin contents High viscosity	N.S.	[38]
	Chemical	Physicochemical Functional	Source of RS Total starch content Water absorption Low glycaemic index	N.S.	[39]
Guava seeds	Chemical	Physicochemical	Amylose and amylopectin contents RS content Digestible starch content	N.S.	[40]
Grape residue meal	Physical/mechanical	Physicochemical	Anthocyanins Fibre	N.S.	[11]
Guinea arrowroot (*Calathea allouia*)	Physical/chemical	Physicochemical Structural	Amylose content RS content Crystallinity	RS3 RS4	[11]
Mango seeds	Physical/chemical	Physicochemical Morphological Structural Thermal	Amylose and amylopectin contents RS content Granular form High crystallinity Higher gelatinisation temperature (78.13–82.39 °C) Enthalpy (17.32–19.45 J/g)	RS2	[12]
Under-ripe banana pulp and peel	Physical/chemical	Physicochemical	Low starch content High dietary fibre RS content	N.S.	[41]
Rice endosperm	Physical	Physicochemical Morphological Structural	Amylose content Polygonal, elongated starch Crystallinity	N.S.	[42]
Soya husk (*Glycine max* L.)	Physical/chemical	Physicochemical	Cellulose	N.S.	[43]
Cacao husk	Physical/chemical	Physicochemical Functional	RS content Antioxidant capacity	N.S.	[44]
Huaya seed *(Melicoccus bijugatu)*	Physical/chemical	Physicochemical Thermal Structural Functional	Starch content RS content Low swelling capacity Amylose content Gelatinisation temperature (81.45 ± 0.15 °C) Crystallinity High viscosity (thickening property)	N.S.	[45]
Melon mask	Physical/chemical	Physicochemical Functional	RS content Dietary fibre Glycaemic effect Starch digestibility	N.S.	[46]
Curcuma root	Chemical/mechanical	Physicochemical Morphological Structural Functional	Amylose content Starch content Swelling power Solubility Crystallinity Phenols Antioxidant activity	N.S.	[47]
Grape mask	Physical/mechanical	Physicochemical	Dietary fibre Phenolic compounds	N.S.	[48]

Note: N.S.: Not specified.

**Table 2:** Key equations and metrics used in the quantitative assessment of risk from exposure to potentially toxic elements (PTEs).

Parameter	Description			Reference
EDI	Estimated daily intake	C · IR/Bw	mg/kgBw/day	[77]
C	Concentration	Potentially toxic element	mg/kg	
IR	Ingestion rate		kg/day	
Bw	Body weight		kgBw	
HQ	Hazard quotient	EDI/RV		[78]
RV	Reference value	RfD; potentially toxic element	mg/kgBw/day	
HI	Hazard index	$\sum_{n=1}^{x}{HQ}_{n}$		[79]
MOE	Margin of exposure	BMDL_%_/EDI		[80]
BMDL_%_	Benchmark dose	BMDL_%_; potentially toxic element		
POE	Probability of exceedance	$Pr\left(EDI>BMDL\right)=\int_{{BMDL}_{\%}}^{\infty }f(E)dE$		
CR	Cancer risk	EDI · SF		[78]
SF	Slope factor	SF; potentially toxic element	(mg/kgBw/day)^−1^	

## Data Availability

The data presented in this study are available on request from the corresponding author.

## Supplementary Materials

The following supporting information can be downloaded at https://www.mdpi.com/article/10.3390/foods14162815/s1, Figure S1: Scientific production per year; Figure S2: Authors with the greatest scientific output on the subject; Figure S3: Affiliations associated with the authors of publications in the field.
