# Supplementary material for: Advances in Resistant Starch Research from Agro-Industrial Waste: A Bibliometric Analysis of Scientific Trends
Source: Foods. 2025 Aug 14;14(16):2815. doi: 10.3390/foods14162815 (PMC12386043; doi:10.3390/foods14162815)
Supplement: Supplementary file 1 [file foods-14-02815-s001.zip › foods-3785139-supplementary.pdf]

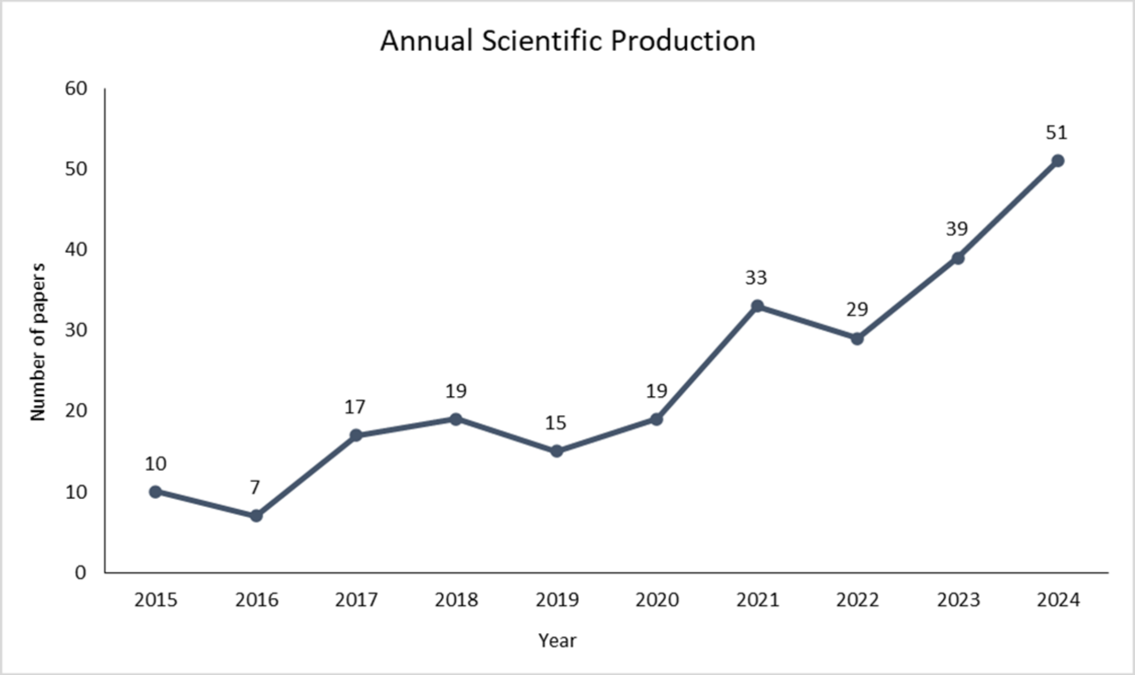

Figure S1. Scientific production per year.

.

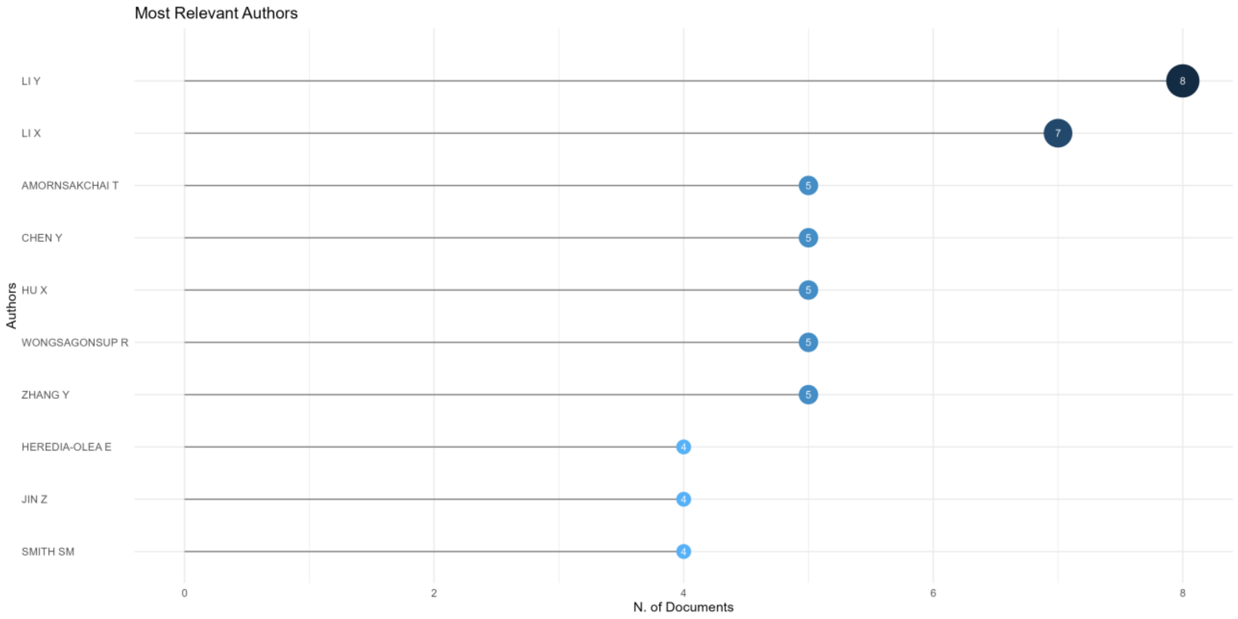

Figure S2. Authors with the greatest scientific output on the subject.

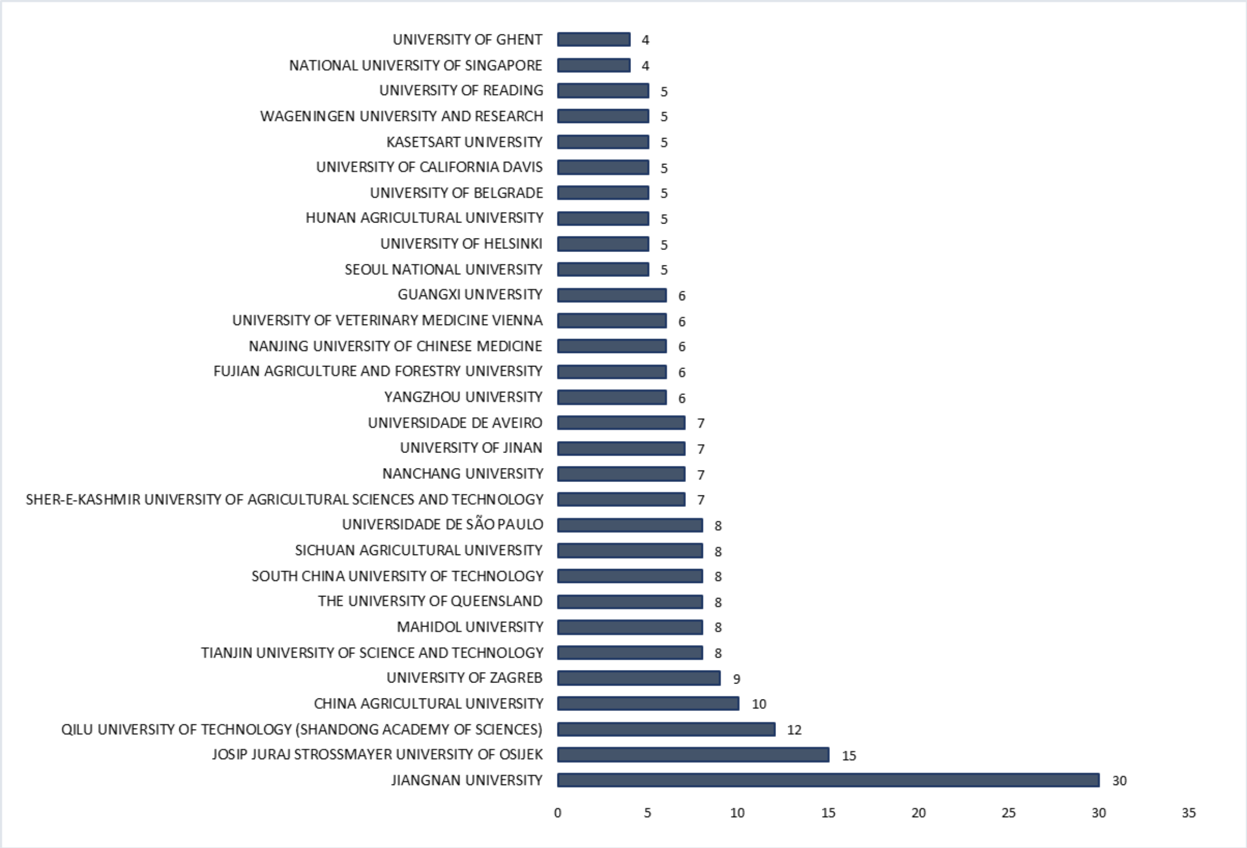

Figure S3. Affiliations associated with the authors of publications in the field.
